# Environmental DNA-Based Bacterial Community Characteristics in Rural Greywater: A Case Study from Eastern China

**DOI:** 10.3390/biology15131069

**Published:** 2026-07-03

**Authors:** Zhenjun Tian, Lieyu Zhang, Shengwang Gao, Yimei Wei, Yangwei Bai, Shuping Wang

**Affiliations:** 1 State Key Laboratory of Environmental Criterion and Risk Assessment, Chinese Research Academy of Environmental Sciences, Beijing 100012, China; tianzj@craes.org.cn (Z.T.);; 2 Key Laboratory of Estuarine and Coastal Environment, Ministry of Ecology and Environment, Beijing 100012, China

**Keywords:** rural greywater, bacterial community, environmental drivers, absolute quantification 16S rRNA

## Abstract

Rural greywater management is a critical global challenge due to the lack of centralized treatment in dispersed communities. This study aimed to characterize the pollution characteristics and bacterial community structure of samples from four greywater collection tanks in eastern China using high-throughput sequencing and absolute quantification of the 16S rRNA gene. Pollution characteristics showed spatial heterogeneity: chemical oxygen demand ranged from 19.8 to 272.5 mg/L, total nitrogen from 8.6 to 16.4 mg/L, and dissolved oxygen from 1.3 to 5.3 mg/L. Dissolved greenhouse gases also varied, with N_2_O reaching 103.6 ppmv and CH_4_ up to 50.4 ppmv. Based on the estimated absolute abundance of 16S rRNA gene copies, we found that the bacterial communities were dominated by Pseudomonadota, Actinomycetota, Bacteroidota, and Bacillota. Key genera such as *Acinetobacter*, *Pseudomonas*, and *unclassified Enterobacteriaceae* were positively correlated with nitrate, suggesting their potential association with denitrification and potential N_2_O production. The methanotrophic genus *Methyloparacoccus* was enriched in a tank with high dissolved organic carbon. Co-occurrence network analysis revealed that core taxa like *unclassified Paracoccaceae* and *Limnohabitans* function as module hubs, maintaining community stability. These findings reveal associations between bacterial taxa, pollutant transformation, and greenhouse gas emissions in rural greywater and provide fundamental insights to support the development of low-carbon, resource-oriented treatment technologies.

## Simple Summary

Managing greywater from rural homes is a growing environmental challenge, especially in countries with large rural populations like China. Unlike cities, rural areas often lack centralized treatment systems, leading to pollution of rivers and soil. This study examined greywater from four rural villages in eastern China to understand its pollution levels and the bacterial communities living in it. These bacteria naturally break down pollutants, but they can also produce greenhouse gases. The results showed that pollution characteristics varied between villages, with some having high levels of organic matter while others had elevated levels of nutrients such as nitrogen. The types and amounts of bacteria also differed, with certain bacteria linked to greenhouse gas production. By understanding which bacteria thrive under different pollution conditions, scientists can design better, more efficient treatment systems for rural areas. This knowledge helps protect local water resources and supports global goals for cleaner, more sustainable rural environments.

## 1. Introduction

The treatment of rural greywater constitutes a critical global challenge for water environmental protection [1]. Worldwide, hundreds of millions of rural residents still lack access to appropriate greywater treatment facilities [2,3]. Taking China as an example, data from the National Bureau of Statistics show that the rural permanent population reached 477 million by the end of 2023, accounting for 33.8% of the national total population. In contrast to densely populated urban areas, rural communities are geographically scattered, making the construction of centralized greywater collection pipelines economically costly and technically difficult. As a result, rural greywater cannot be effectively collected and treated using urban centralized treatment models [4]. A large volume of rural greywater is discharged directly or after only simple pretreatment, triggering severe environmental problems such as surface water eutrophication, soil degradation, and the spread of pathogenic bacteria [5]. Therefore, the scientific management of rural greywater is not only a key strategy for improving rural living environments and residents’ quality of life, but also an indispensable pathway for achieving sustainable water resource utilization and aquatic ecological conservation at a global scale.

Notably, rural greywater is defined as household wastewater generated from kitchens, laundry, cleaning, bathing, and yard washing, excluding toilet blackwater, and is characterized by relatively low pollutant loads [6]. Recent studies have demonstrated that greywater is characterized by low ammonia nitrogen (NH_4_^+^-N) and total phosphorus (TP) concentrations, moderate-to-high chemical oxygen demand (COD), and low public health risks [3,7]. Such greywater can be efficiently purified via suitable decentralized treatment technologies, and the reclaimed effluent has the potential to be reused for agricultural irrigation, landscape greening, and other resource-recycling purposes [8,9]; however, the present study focuses solely on the characterization of raw greywater. The systematic management of rural greywater is therefore of great significance for protecting rural water environmental security, maintaining regional aquatic ecosystem health, and promoting sustainable development in extensive rural regions [10].

Rural greywater contains indigenous bacterial communities that not only mediate the transformation of carbon, nitrogen, phosphorus, and other nutrients, but also impose non-negligible effects on the performance of treatment technologies and the ecology of receiving water bodies [11,12]. Nevertheless, the current understanding of the community structure, diversity, and ecological functions of inherent bacteria in rural greywater remains insufficient. Key scientific issues, including how these bacteria synergistically drive organic matter degradation, nutrient conversion, and greenhouse gas production and emission under anaerobic, aerobic, and anoxic conditions, are still poorly understood [13]. This knowledge gap has directly restricted the rational selection and precise optimization of resource-oriented rural greywater treatment processes [3]. Without in-depth insights into carbon and nitrogen metabolic pathways and greenhouse gas emission patterns during biological treatment, it is difficult to optimize operational parameters to realize high-efficiency water purification while controlling greenhouse gas emissions. Accordingly, systematically revealing the bacterial taxonomic composition inferred from environmental DNA, along with functional traits and environmental response mechanisms in rural greywater, is a prerequisite for developing low-carbon and resource-recycling treatment technologies and is critically important for achieving carbon peaking and carbon neutrality goals in rural greywater management.

Against the above background, this study collected greywater samples from centralized collection tanks at four typical rural sites in eastern China. Using high-throughput sequencing of 16S rRNA genes, we analyzed the bacterial community characteristics of the samples. The aim was to elucidate the bacterial community features of rural greywater, offering fundamental data and theoretical support for the future development and optimization of low-carbon treatment processes.

## 2. Materials and Methods

### 2.1. Study Area and Sample Collection

**Figure 1:**
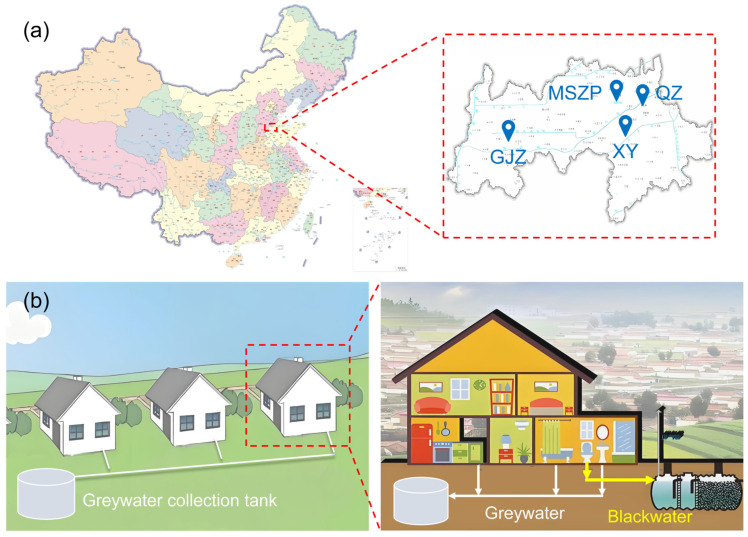
Schematic diagram of study area (**a**) and greywater source (**b**).

As shown in Figure 1a, the study area is located in Shandong Province, China, covering approximately 117.28° E–117.74° E and 37.52° N–37.69° N. Four centralized collection tanks for rural greywater were selected for sampling, and detailed information on these tanks is provided in Table 1. As illustrated in Figure 1b, these collection tanks receive only greywater from household washing and cleaning activities, including wastewater from the kitchen (after grease separation), handwashing basins, showers, and washing machines. Wastewater from toilets is excluded as it is separately collected into septic tanks. All sampling was conducted in April, and only a single sampling event was performed without replicates; therefore, the results represent a temporal snapshot. A clear limitation is the small sample size without replicates, which was due to the exploratory nature of this study. Analytical thresholds for all parameters followed the APHA standard methods [14].

### 2.2. Determination of Greywater Composition

Immediately after collection, water samples were measured for temperature (T), dissolved oxygen (DO), and pH using a multifunctional water-quality device (Multi 3620 IDS, WTW, Germany). A 5 L water sample was transported to the laboratory in an incubator at −4 °C, and other physical and chemical parameters were analyzed within 24 h. Conductivity and total dissolved solids (TDS) were measured using a portable multiparameter analyzer (SL 1000, HACH, USA). Suspended solids (SS), COD, NH_4_^+^-N, nitrate nitrogen (NO_3_^−^-N), total nitrogen (TN), and TP were determined following standard protocols for water and wastewater [14]. The concentrations of dissolved nitrous oxide (N_2_O), carbon dioxide (CO_2_), and methane (CH_4_) in the four rural greywater collection tanks were determined using the headspace-gas chromatography method with a gas chromatograph (Agilent 7890B, USA).

### 2.3. Microbial Sequencing

We characterized the bacterial community composition and estimated absolute abundance of 16S rRNA gene copies in rural greywater collection tanks using a high-throughput sequencing approach based on absolute quantification of the 16S rRNA gene. This method allowed an accurate assessment of bacterial community structure and variations across different collection tanks. Absolute quantification was achieved by spiking each sample with synthetic DNA sequences (spike-in DNA) at known concentration gradients. These exogenous sequences were co-amplified and sequenced together with the sample-derived DNA. A standard curve was constructed based on the known copy numbers of the spike-in DNA and their corresponding sequencing reads, enabling calculation of the estimated absolute abundance of 16S rRNA gene copies assigned to each taxon in the sample. All experimental procedures, including DNA extraction, PCR amplification, sequencing, and raw data quality control, were strictly followed according to the protocol established in our previous study [15].

### 2.4. Statistical Analysis

A phylogenetic tree of the top 20 genera by absolute abundance was constructed using the Maximum Likelihood method [16]. To explore relationships between bacterial communities and environmental factors, Spearman’s rank correlation coefficient was calculated between the top 15 genera by absolute abundance and the measured environmental variables [17]. Coexistence patterns among bacterial taxa at the genus level were analyzed by constructing a co-occurrence network using the Python package networkx (version 2.7). Furthermore, a single-factor correlation network was developed using R software (version 3.3.1, stat package) and Python (version 2.7, stat module) to investigate species–species interactions among the top 50 genera by absolute abundance [18]. Significant correlations among the genera (|R| > 0.7, *p* < 0.05) were defined by Spearman’s correlation coefficient.

## 3. Results and Discussion

### 3.1. Environmental Variables of Rural Greywater

#### 3.1.1. Pollution Characteristics

As shown in Table 2, the physicochemical characteristics of the four rural greywater collection tanks are marked by uneven pollution levels. T ranged from 23.1 to 26.2 °C, all falling within a range suitable for bacterial activity [19]. Regarding DO, the GJZ and QZ tanks recorded values of only 1.31 mg/L and 1.27 mg/L, respectively, indicating hypoxic conditions. In contrast, the XY and MSZP tanks showed relatively better oxygen conditions, with DO values of 4.79 mg/L and 5.27 mg/L, suggesting that the former two tanks may be subject to higher oxygen-consuming pollution loads or poorer water circulation. The pH values ranged from 7.42 to 8.57, with all samples being slightly alkaline to alkaline; the XY tank exhibited the strongest alkalinity (pH 8.57). SS concentrations ranged from 12 to 56 mg/L, with the XY tank recording the highest value and the QZ tank the lowest, indicating differences in particulate matter input intensity across the tanks. TCOD varied widely: the GJZ tank had a total COD (TCOD) of only 19.75 mg/L, while the XY, MSZP, and QZ tanks reached 272.50 mg/L, 197.50 mg/L, and 179.75 mg/L, respectively, indicating that apart from GJZ, the other three tanks were subject to severe organic pollution. Further analysis of the soluted COD (SCOD) proportion showed that the SCOD/TCOD ratios in the XY and QZ tanks exceeded 90%, suggesting that organic matter in these tanks was predominantly in dissolved form, whereas the MSZP tank had a SCOD/TCOD ratio of approximately 49%, indicating comparable contributions from particulate and dissolved organic matter. In terms of nitrogen species, NH_4_^+^-N was highest in the MSZP tank (11.06 mg/L), significantly exceeding the levels in the other tanks (0.06–5.25 mg/L). In contrast, NO_3_^−^-N showed an opposite trend, with higher concentrations in the GJZ and QZ tanks (4.19 mg/L and 6.53 mg/L, respectively) and lower concentrations in the XY and MSZP tanks (≤0.70 mg/L), reflecting differences in nitrification intensity or nitrogen transformation pathways among the tanks. TN was highest in the MSZP tank (16.39 mg/L), while the other tanks ranged from 8.6 to 10.99 mg/L. TP concentrations were generally low, ranging from 0.44 to 0.79 mg/L, with no evidence of significant phosphorus enrichment. Overall, the XY, MSZP, and QZ tanks exhibited varying degrees of organic matter and nitrogen pollution, with the MSZP tank characterized by high NH_4_^+^-N, while the GJZ tank showed relatively low pollution levels but insufficient DO. These results highlight that the pollution characteristics of rural greywater exhibited heterogeneity.

#### 3.1.2. Greenhouse Gas Emission Potential

The concentrations of dissolved greenhouse gases in the four rural greywater collection tanks exhibited spatial heterogeneity (Table 3), which was closely associated with the pollution characteristics of each tank. Tank QZ showed the highest N_2_O and CO_2_ concentrations (103.6 ppmv and 9231.6 ppmv, respectively). The combination of low DO (1.27 mg/L), high NO_3_^−^-N (6.53 mg/L), and a high proportion of SCOD (>90%) in QZ favored N_2_O accumulation during denitrification. In contrast, although tank GJZ also exhibited low DO (1.31 mg/L) and high NO_3_^−^-N (4.19 mg/L), its extremely low TCOD (19.75 mg/L) likely limited the electron donor supply for denitrification, resulting in a much lower N_2_O concentration (1.3 ppmv). The distribution of CH_4_ showed an opposite pattern, with significantly higher concentrations in GJZ and MSZP (45.3 and 50.4 ppmv, respectively) than in XY and QZ (0.2 and 0.3 ppmv, respectively). The elevated CH_4_ levels in GJZ and MSZP were attributed to hypoxic conditions (GJZ) or the formation of anoxic microzones under high organic loading (MSZP), whereas the higher DO in XY (4.79 mg/L) and the high NO_3_^−^-N in QZ suppressed methanogenesis. Collectively, these results indicate that DO, organic carbon availability and forms, and nitrogen transformation pathways collectively regulate the production and accumulation of greenhouse gases in rural greywater [20]. Therefore, assessing the emission potential of greenhouse gases from such collection systems requires process-based analysis incorporating site-specific pollution characteristics.

### 3.2. Bacterial Community Characteristics of Rural Greywater

#### 3.2.1. Composition of Bacterial Community

**Figure 2:**
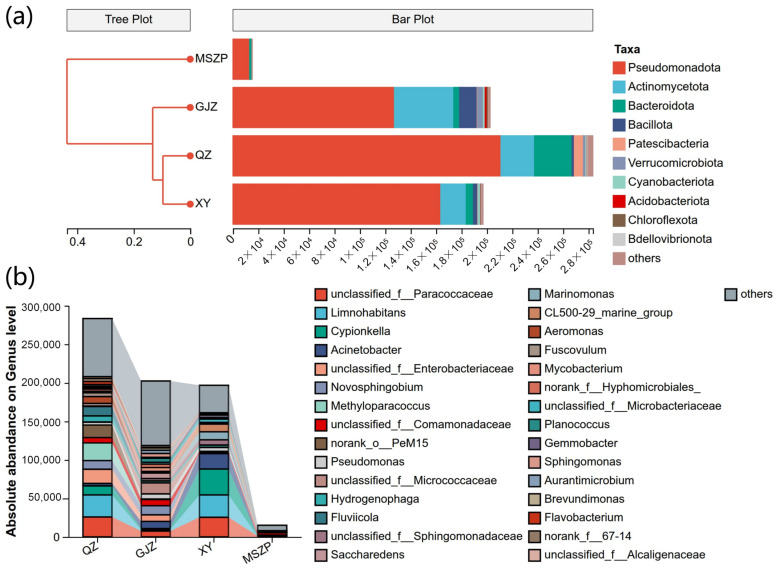
Bacterial community characteristics: (**a**) Hierarchical clustering tree on phylum level and (**b**) composition of community on genus level.

To investigate the estimated absolute abundance of 16S rRNA gene copies and the community characteristics of bacteria in four rural greywater collection tanks, we employed a high-throughput sequencing method based on absolute quantification of the 16S rRNA gene, enabling precise characterization of bacterial community composition and variations across different tanks. Hierarchical clustering analysis at the phylum level (Figure 2a) revealed that the bacterial community structures of the four rural greywater collection tanks could be divided into two main clusters. Specifically, QZ and XY first clustered together, followed by GJZ, while MSZP formed a separate, distinct branch. This clustering pattern indicates that the bacterial community compositions of QZ, XY, and GJZ are relatively similar, whereas the community structure of MSZP is markedly different. In terms of dominant phyla, the taxa with relatively high estimated abundance of 16S rRNA gene copies across all samples included Pseudomonadota (formerly Proteobacteria), Actinomycetota, Bacteroidota, and Bacillota (formerly Firmicutes), which are widely involved in organic matter degradation, nitrogen cycling, and fermentation processes [21,22,23]. Additionally, other phyla such as Patescibacteria, Verrucomicrobiota, Cyanobacteriota, Acidobacteriota, Chloroflexota, and Bdellovibrionota were also detected but at relatively low estimated abundances. The differences in community composition at the phylum level among the collection tanks reflect the shaping effects of varying environmental conditions on bacterial community structure [24]. The clear separation of MSZP from the other three tanks in the clustering analysis suggests that it possesses a unique dominant bacterial community structure. Although QZ, XY, and GJZ were grouped into the same major cluster, the clustering distances among them still exhibited differences, indicating a certain degree of inter-sample variation in their phylum-level community compositions.

Based on the estimated absolute abundance of 16S rRNA gene copies at the genus level (Figure 2b), the bacterial community compositions of the four rural greywater collection tanks exhibited differences. In tank QZ, the genera with relatively high estimated abundance included *unclassified_f_Paracoccaceae*, *Limnohabitans*, *Cypionkella*, *Acinetobacter*, and *unclassified_f_Enterobacteriaceae*. Among these, certain members of *Acinetobacter* and *Enterobacteriaceae* possess denitrification functions [25,26], consistent with the high N_2_O concentration (103.6 ppmv) observed in QZ, suggesting that these taxa may be the main contributors to incomplete denitrification. The community composition of tank GJZ was similar to that of QZ, and was also enriched in the above-mentioned dominant genera. Additionally, the estimated abundance of *Pseudomonas* was higher in GJZ; this genus contains various species capable of organic matter degradation and denitrification [27]. The low DO level and high CH_4_ concentration in GJZ further suggest the presence of anoxic microenvironments that support methanogens, although typical methanogenic archaea were not directly detected in the genus-level dataset (as it focuses on bacteria). Tank XY exhibited a somewhat different composition of dominant genera compared to the other three tanks. Although *unclassified_f_Paracoccaceae*, *Limnohabitans*, and *Cypionkella* were also present, the relative estimated abundance of *Acinetobacter* was relatively low. The higher DO (4.79 mg/L) and pH (8.57) in XY may have inhibited the enrichment of certain anaerobic or denitrifying groups, consistent with its low N_2_O and CH_4_ concentrations. Tank MSZP displayed the most unique community structure at the genus level. Its dominant genera included *unclassified_f_Paracoccaceae*, *Limnohabitans*, and *Cypionkella*, while the estimated abundance of *Methyloparacoccus* was significantly higher than in the other three tanks. *Methyloparacoccus*, a genus of methanotrophs, may be involved in methane consumption [28]. This is not contradictory to the observed high CH_4_ concentration (50.4 ppmv) in MSZP despite its relatively high DO level (5.27 mg/L), suggesting that methane production and oxidation may occur simultaneously. Furthermore, the enrichment of taxa such as *norank_o_PeM15* in MSZP warrants further investigation regarding their ecological functions. In summary, the bacterial community compositions at the genus level exhibited spatial heterogeneity among the four rural greywater collection tanks. The community structures of QZ and GJZ were relatively similar, whereas MSZP displayed unique dominant taxa. These differences are closely related to the pollution characteristics and greenhouse gas production potential of each tank.

**Figure 3:**
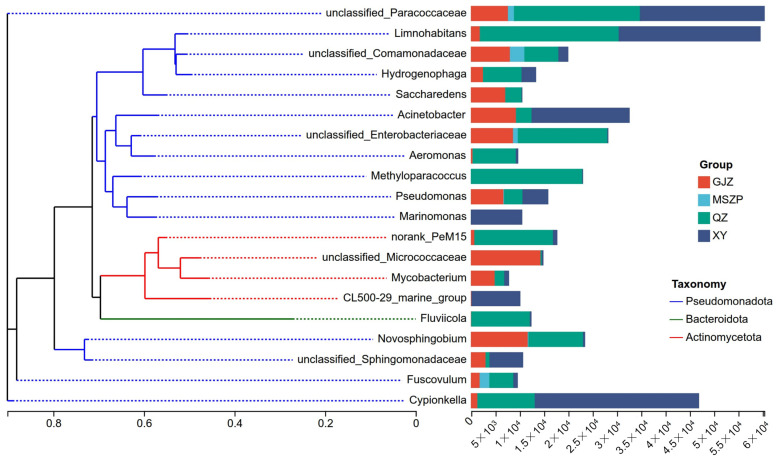
Phylogenetic tree on genus level.

A phylogenetic tree constructed at the genus level (Figure 3) illustrates the evolutionary relationships among the dominant taxa detected in the four rural greywater collection tanks. The tree comprises 20 representative genera, primarily belonging to three phyla: Pseudomonadota, Bacteroidota, and Actinomycetota. Among these, the genera affiliated with Pseudomonadota include *unclassified_f_Paracoccaceae*, *Limnohabitans*, *unclassified_f_Comamonadaceae*, *Hydrogenophaga*, *Acinetobacter*, *unclassified_f_Enterobacteriaceae*, *Aeromonas*, *Methyloparacoccus*, *Pseudomonas*, *Marinomonas*, Novosphingobium, *unclassified_f_Sphingomonadaceae*, *Fuscovulum*, and *Cypionkella*, reflecting the dominance of these genera across all samples. The phylum Bacteroidota is represented by *Fluviicola* and *Saccharodendrons*, while Actinomycetota includes *unclassified_f_Micrococcaceae*, *Mycobacterium*, and *CL500-29_marine_group*.

The phylogenetic tree shows that genera within the same phylum generally cluster together, consistent with their taxonomic positions. Notably, the methanotrophic genus *Methyloparacoccus* is positioned within the Pseudomonadota branch, while the denitrifying genera *Acinetobacter* and *Pseudomonas* are also located in distinct but related branches. Furthermore, certain genera (e.g., *unclassified_f_Paracoccaceae* and *Limnohabitans*) exhibit relatively close evolutionary distances, suggesting potential functional similarities or ecological co-occurrence [29]. Overall, this genus-level phylogenetic tree provides a taxonomic framework for understanding the evolutionary relationships among dominant bacterial lineages in rural greywater collection systems and supports further functional predictions based on phylogenetic placement.

#### 3.2.2. Correlation Between Bacterial Community and Environmental Factors

**Figure 4:**
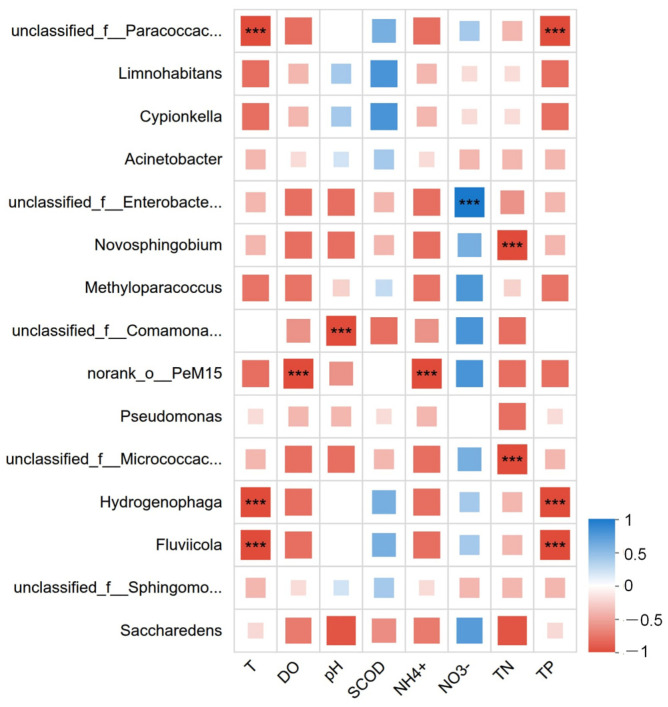
Spearman correlation heatmap between bacterial community and environmental factors. (*** *p* ≤ 0.001).

Based on Spearman correlation analysis, the relationships between 12 dominant genera and environmental factors in the four rural greywater collection tanks were evaluated (Figure 4). The results showed that some typical bacteria were significantly (*p* ≤ 0.001) affected by environmental variables. *unclassified_f_Paracoccaceae*, *Hydrogenophaga*, and *Fluviicola* showed significant correlations with T and TP (*p* ≤ 0.001), suggesting their potential involvement in phosphorus metabolism in greywater. *norank_o_PeM15* was positively correlated with DO and NH_4_^+^-N (*p* ≤ 0.001), implying its possible involvement in nitrification [30]. Overall, the correlation patterns between dominant genera and environmental factors are generally consistent with their potential ecological functions, further supporting the selective effects of environmental variables on bacterial community structure and their potential regulation of greenhouse gas production pathways [31]. Given the limited sample size, these correlations should be interpreted as exploratory hypotheses rather than definitive ecological conclusions, and further validation with larger datasets is required.

#### 3.2.3. Characteristics of Bacterial Network

**Figure 5:**
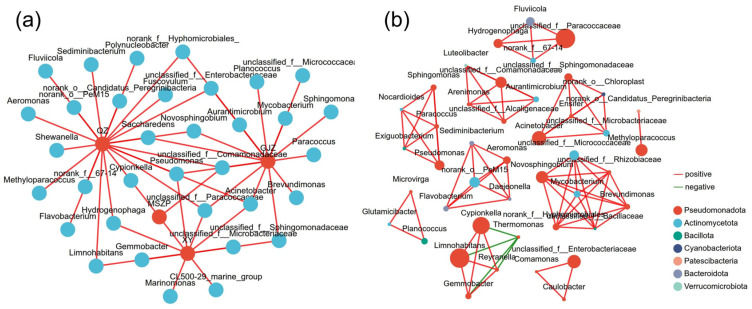
Bacterial networks of rural greywater: (**a**) Co-occurrence network shows the distribution between samples and species, (**b**) Single factor correlation network shows the correlation between species.

As shown in Figure 5, co-occurrence network analysis of bacterial communities from four rural greywater collection tanks revealed an association pattern dominated by a few keystone taxa. Node attributes indicated that *unclassified_f_Paracoccaceae*, *Limnohabitans*, *Cypionkella*, *Acinetobacter*, and *Pseudomonas* exhibited high degree and weighted degree centrality, positioning them at the network core. These taxa likely act as module hubs connecting different functional groups and play a critical role in maintaining community structural stability [32]. From an ecological perspective, these core nodes are predominantly heterotrophic degraders (e.g., *Limnohabitans*, *Acinetobacter*, *Pseudomonas*) or nitrogen-cycling-related bacteria (e.g., *Hydrogenophaga*, *unclassified_f_Paracoccaceae*), and their high connectivity reflects a tight coupling between organic matter mineralization and nitrogen transformation processes within the community [33,34,35]. From the perspective of network stability, the widespread presence of highly connected core taxa enhances community resistance to environmental perturbations [36]. Moreover, multiple module hubs, such as *unclassified_f_Paracoccaceae* and *Limnohabitans*, connect different metabolic functional groups, forming functionally complementary co-occurrence modules. This modular architecture helps maintain the continuity of key metabolic pathways under fluctuating influent quality, which is a common characteristic of rural greywater collection tanks, thereby enhancing the overall ecological stability of the bacterial community.

The single-factor correlation network focuses on significant association patterns among dominant microbial genera in four rural greywater collection tanks. All correlation coefficients in the network are either +1 or −1, indicating extremely tight and highly structured node associations. Node size represents species abundance, with *Novosphingobium*, *unclassified_f_Rhizobiaceae*, *norank_f_Hyphomicrobiales*, *Brevundimonas*, *unclassified_f_Bacillaceae*, *unclassified_f_Micrococcaceae*, and *Mycobacterium* exhibiting both large node sizes and high degree values. This indicates that these species are not only abundant but also participate in extensive, strong positive correlations, positioning them as core hub nodes. From the phylum-level coloring, these high-abundance, high-connectivity nodes primarily belong to Pseudomonadota and Actinomycetota, suggesting that these two phyla dominate the wastewater bacterial community and form tight synergistic relationships with dominant species from other phyla, such as Bacteroidota and Bacillota [37].

Multiple nodes in the network form fully connected subgraphs (cliques), where any two nodes within a subgraph exhibit significant positive correlations with thick edges (*p* < 0.05). For example, the seven nodes including *Novosphingobium* are all pairwise positively correlated, forming a highly tight functional module. These species originate from Pseudomonadota, Actinomycetota, and Bacillota, indicating that dominant species from different phyla can form cross-phylum strong synergistic relationships. Similarly, *Aeromonas*, *Flavobacterium*, *Sediminibacterium*, *Daejeonella*, and *norank_o_PeM15* constitute another fully connected subgraph, suggesting potential metabolic complementarity between Bacteroidota, Pseudomonadota, and Patescibacteria in organic matter degradation [38,39]. Negative correlations (green edges) are also present in the network, primarily between *Thermomonas* and *Limnohabitans*, *Gemmobacter*, *Reyranella*, and *Cypionkella*, with a coefficient of −1. Notably, these five nodes themselves are all pairwise positively correlated with each other, forming a competitive module characterized by “internal synergy and external exclusion”. *Thermomonas* is a thermophilic or thermotolerant genus [40], while *Limnohabitans* and related genera are typically mesophilic [41]. The strong negative correlations likely reflect niche differentiation due to differences in temperature adaptation or substrate competition. From the overall network structure, all nodes have a clustering coefficient of 1, indicating that the network consists of multiple fully connected subgraphs without isolated nodes. Node size shows a positive trend with degree, meaning that more abundant species tend to participate in more significant correlations, consistent with the ecological principle of “dominant species as network cores” [42]. In the context of fluctuating influent quality in rural greywater collection tanks, this highly modular network structure may represent an adaptive strategy: core functional groups maintain stable operation of key metabolic processes through strong internal synergies, while negative correlations between different functional groups promote niche differentiation and reduce direct competition for overlapping functions [43]. In summary, this network reveals a highly structured association pattern characterized by Pseudomonadota and Actinomycetota as core hubs, with cross-phylum strong synergies coexisting with inter-module exclusion, providing important insights into the interaction mechanisms of dominant species in wastewater ecosystems. However, due to the limited sample size, the observed extreme correlation coefficients may overestimate the strength of ecological interactions. Therefore, the network patterns reported here are preliminary and should be verified with larger sample sets before drawing strong ecological conclusions.

## 4. Conclusions

This study systematically characterized the bacterial community in rural greywater collected from four typical villages in eastern China using the absolute quantification of 16S rRNA gene sequencing. The results showed that bacterial communities were consistently dominated by Pseudomonadota, Actinomycetota, Bacteroidota, and Bacillota. However, the estimated absolute abundances of key functional genera varied among the sites. These included the denitrifying genera *Acinetobacter* and *Pseudomonas*, as well as the methanotrophic genus *Methyloparacoccus*. Correlation and network analyses further demonstrated that DO and dissolved organic carbon act as master variables shaping both greenhouse gas production pathways and the co-occurrence patterns of core bacterial taxa. Overall, these findings provide critical baseline data and theoretical support for understanding the microbial mechanisms driving carbon and nitrogen transformation in rural greywater and for developing low-carbon treatment processes. However, as this study did not assess pathogenic microorganisms or health-related indicators (e.g., fecal coliforms, *Escherichia coli*), the results do not support conclusions regarding the safety of greywater reuse for irrigation or other resource-oriented applications. Future studies should incorporate health-risk assessments, including the monitoring of key pathogens and opportunistic pathogens, to comprehensively evaluate the suitability of treated greywater for irrigation and other reuse purposes.

## Figures and Tables

**Table 1:** Information on rural greywater collection tanks.

Samples	Gujiazhai (GJZ)	Xiangyang (XY)	Maosaizhenpai (MSZP)	Qianzhang (QZ)
Number of households covered (household)	24	58	117	162
Service population (person)	78	216	415	438
Volume of collection tank (m^3^)	10	40	30	40

**Table 2:** Pollution characteristics of rural greywater.

Samples	GJZ	XY	MSZP	QZ
Temperature (°C)	25.6 ± 0.1	24.2 ± 0.1	26.2 ± 0.1	23.1 ± 0.1
DO (mg/L)	1.31 ± 0.04	4.79 ± 0.02	5.27 ± 0.06	1.27 ± 0.13
pH	7.42 ± 0.08	8.57 ± 0.03	8.01 ± 0.02	7.80 ± 0.03
SS (mg/L)	46 ± 2	56 ± 1	44 ± 3	12 ± 3
TCOD (mg/L)	19.75 ± 2.44	272.50 ± 12.63	197.50 ± 32.04	179.75 ± 16.24
SCOD (mg/L)	4.75 ± 1.06	247.50 ± 32.11	97.5 ± 22.53	169.75 ± 12.05
NH_4_^+^-N (mg/L)	0.09 ± 0.02	5.25 ± 0.06	11.06 ± 1.06	0.06 ± 0.01
NO_3_^−^-N (mg/L)	4.19 ± 1.25	0.43 ± 0.12	0.70 ± 0.24	6.53 ± 0.51
TN (mg/L)	8.60 ± 0.73	10.99 ± 1.16	16.39 ± 1.65	10.22 ± 1.03
TP (mg/L)	0.68 ± 0.08	0.59 ± 0.25	0.79 ± 0.62	0.44 ± 0.25

**Table 3:** Content of dissolved greenhouse gases in rural greywater.

Samples	GJZ	XY	MSZP	QZ
N_2_O (ppmv)	1.3 ± 0.3	0.5 ± 0.2	0.1 ± 0.0	103.6 ± 2.2
CO_2_ (ppmv)	2613.4 ± 121.2	1535.0 ± 88.4	4192.6 ± 228.3	9231.6 ± 108.4
CH_4_ (ppmv)	45.3 ± 0.7	0.2 ± 0.1	50.4 ± 1.3	0.3 ± 0.1

## Data Availability

Data are available upon reasonable request.
